# Photothermally Triggered Endosomal Escape and Its Influence on Transfection Efficiency of Gold-Functionalized JetPEI/pDNA Nanoparticles

**DOI:** 10.3390/ijms19082400

**Published:** 2018-08-14

**Authors:** Lotte M. P. Vermeulen, Juan C. Fraire, Laurens Raes, Ellen De Meester, Sarah De Keulenaer, Filip Van Nieuwerburgh, Stefaan De Smedt, Katrien Remaut, Kevin Braeckmans

**Affiliations:** 1 Laboratory of General Biochemistry & Physical Pharmacy, Ghent University, Ottergemsesteenweg 460, 9000 Ghent, Belgium; lottem.vermeulen@ugent.be (L.M.P.V.); Juan.Fraire@ugent.be (J.C.F.); laurens.raes@ugent.be (L.R.); stefaan.desmedt@ugent.be (S.D.S.); katrien.remaut@ugent.be (K.R.); 2 Centre for Nano- and Biophotonics, Ghent University, Ottergemsesteenweg 460, 9000 Ghent, Belgium; 3 Laboratory of Pharmaceutical Biotechnology, Ghent University, Ottergemsesteenweg 460, 9000 Ghent, Belgium; Ellen.Demeester@ugent.be (E.D.M.); sarah.Dekeulenaer@ugent.be (S.D.K.); filip.Vannieuwerburgh@ugent.be (F.V.N.); 4 IEMN UMR 8520 and Laboratoire de Physique des Lasers, Atomes et Molécules. UMR 8523, Université de Lille, F−59655 Villeneuve d’Ascq CEDEX, France

**Keywords:** gold nanoparticles, plasmonic effects, endosomal escape, pDNA delivery

## Abstract

Plasmonic nanoparticles for drug delivery have attracted increasing interest over the last few years. Their localized surface plasmon resonance causes photothermal effects on laser irradiation, which allows for delivering drugs in a spatio-temporally controlled manner. Here, we explore the use of gold nanoparticles (AuNP) as carriers for pDNA in combination with pulsed laser irradiation to induce endosomal escape, which is currently considered to be one of the major bottlenecks in macromolecular drug delivery on the intracellular level. In particular, we evaluate nanocomplexes composed of JetPEI (polyethylenimine)pDNA and 10 nm AuNP, which do not exhibit endosomal escape by themselves. After incubating HeLa cells with these complexes, we evaluated endosomal escape and transfection efficiency using low- and high-energy laser pulses. At low laser energy heat is produced by the nanocomplexes, while, at higher laser energy, explosive vapour nanobubbles (VNB) are formed. We investigated the ability of heat transfer and VNB formation to induce endosomal escape and we examine the integrity of pDNA cargo after inducing both photothermal effects. We conclude that JetPEI/pDNA/AuNP complexes are unable to induce meaningful transfection efficiencies because laser treatment causes either dysfunctionality of the cargo when VNB are formed or forms too small pores in the endosomal membrane to allow pDNA to escape in case of heating. We conclude that laser-induced VNB is the most suitable to induce effective pDNA endosomal escape, but a different nanocomplex structure will be required to keep the pDNA intact.

## 1. Introduction

**Figure 1:**
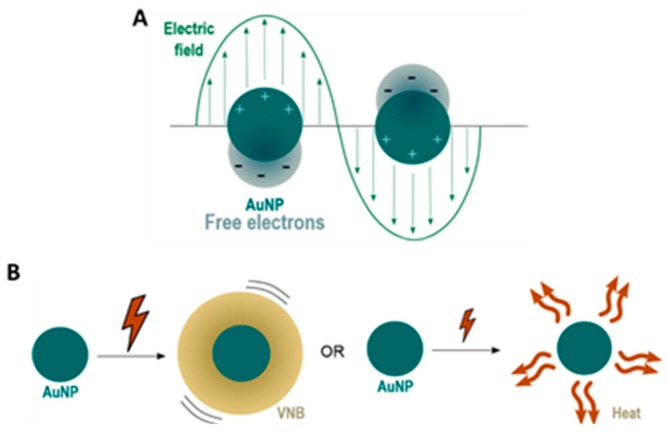
(**A**) under the influence of an external oscillating electromagnetic field, usually provided by laser light, the free electrons of gold nanoparticles (AuNPs) start to oscillate. When the amplitude of this oscillation is maximal, this phenomenon is referred to as Localized Surface Plasmon Resonance (LSPR); (**B**) irradiation of AuNPs with nanosecond laser pulses can lead to the formation of water vapour nanobubbles (VNBs) or heat transfer to the surrounding environment, depending on whether high or low intensity laser pulses are used.

Gold nanoparticles (AuNPs) have attracted increasing interest as a promising new vehicle for gene therapy, stimulated by their basic physical, chemical and optical properties [1]. First of all, the gold core is essentially inert and non-toxic [2]. Secondly, synthesis and surface modifications are fairly straightforward and allow the preparation of a variety of AuNPs that are able to bind macromolecular therapeutics (e.g., proteins, siRNA, pDNA, etc.) [3]. Finally, their optical properties make it interesting to investigate the use of AuNPs for spatio-temporal controlled delivery of the cargo. AuNPs and other metal NPs (e.g., Ag [4,5], Cu [6] and Al [7]) are known to have an enhanced optical absorption via Localized Surface Plasmon Resonance (LSPR). LSPR is a consequence of the interaction between the free electrons of the conduction band of a metal NP and an external oscillating electromagnetic field, as shown in Figure 1A [8,9,10].

Due to the oscillations of the localized surface plasmons, a series of sequential energy transfer processes occur within the NP, which results in an increase of the NP temperature, which is followed by heat transfer from the particle to the environment [11]. By using continuous wave laser irradiation or low intensity laser pulses, direct heat transfer can lead to an increase in temperature of the local surroundings by ten to several hundreds of degrees. On the other hand, the use of intense short laser pulses (<10 ns) can cause an extremely rapid increase in the NP temperature of several hundreds to even thousand degrees. Due to these very high AuNP temperatures, the water surrounding the NP quickly evaporates, leading to the formation of (laser-induced) water vapour nanobubbles (VNB). The expansion and collapse of VNB creates high pressure shockwaves without transferring heat to the environment since nearly all incident laser energy is essentially converted into mechanical energy of the expanding VNB. The size of VNBs can be tuned from tens to several hundreds of nm by varying the laser intensity and the size of NPs [8]. Both heat transfer and VNB formation are depicted in Figure 1B.

LSPR-induced photothermal heating can be used in photothermal therapies. Photothermal cancer therapy is one of the earliest studied applications, where plasmonic NPs act as a localized source of heat to damage and destroy cancer cells [1,9,11]. Later on, these light-triggered properties were employed for drug delivery purposes. Besides the delivery of chemical payloads [12,13,14], currently there is an increasing interest in the use of light-triggered delivery of nucleic acids (NAs) for gene therapy where plasmonic NPs are used as NA nanocarriers. Plasmonic effects can be used for two purposes: (1) light-triggered release of the NA payload at the desired time and place, and (2) overcoming intracellular (IC) barriers. For photothermal release of NAs from plasmonic carriers, different strategies have been investigated to couple NAs to the surface of the NP, which can be subdivided into covalent vs. non-covalent approaches [15]. When the NA is covalently attached to the gold surface, femtosecond laser pulses that break the covalent bond can be employed in order to release the intact NA [16,17]. The second strategy consists of loading the NA onto a carrier molecule using weaker, non-covalent bonds that can be disrupted more easily using lower laser power densities. The carrier molecule should be covalently attached to the gold surface before loading the NA [15,18,19]. Photothermal effects can also aid in tackling IC barriers that obstruct the way for efficient gene delivery. In one approach, especially for in vitro or ex vivo cell transfections, NP-sensitized photoporation of the plasma membrane has been investigated to gain direct access to the cytosol (i.e., without the need to escape endosomes) [20].

**Figure 2:**
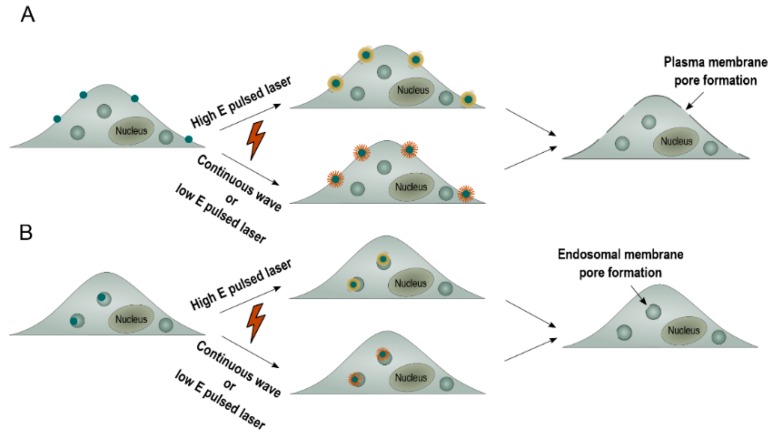
The application of plasmonic NPs to overcome intracellular barriers. (**A**) NP-sensitized plasma membrane photoporation. Cells are incubated with plasmonic NPs to allow attachment of the NPs to the plasma membrane. Next, laser irradiation causes the formation of VNBs (high energy pulsed laser) or heating (continuous wave or low energy pulsed laser). The generation of these plasmonic effects causes the formation of a pore in the plasma membrane, which allows entry of exogeneous compounds into the cell by diffusion; (**B**) light-triggered endosomal escape. Plasmonic NPs are allowed to be taken up by the cell through endocytosis. Next, laser irradiation causes the formation of VNBs (high energy pulsed laser) or heating (continuous wave or low energy pulsed laser). The generation of these plasmonic effects causes the formation of a pore in the endosomal membrane, which allows release of endocytosed cargo.

This so-called photoporation of the plasma membrane can be achieved by thermal membrane permeabilization through a local phase transition of the lipid bilayer in response to the heat transfer of the irradiated plasmonic NP to the environment. Alternatively, the generation of a VNB can cause mechanical membrane poration. In either of both cases, after laser irradiation, exogenous compounds can diffuse through the pores into the cytoplasm (Figure 2A) [8,11]. While this has proven to work very well for compounds up to about a few hundred kDa, delivering larger (charged) molecules such as mRNA or pDNA through the plasma membrane has proven to be more difficult likely due to the pores not being large enough [20,21,22]. An alternative strategy is to allow cellular uptake of NA-loaded plasmonic NPs via endocytosis, followed by light-triggered endosomal escape, as depicted in Figure 2B [23,24]. Plasmonic NPs have already been used successfully to induce light-triggered endosomal rupture and cytosolic delivery of several macromolecules such as proteins [25,26,27], siRNA [15,28,29] and ONs (oligonucleotides) [15]. However, to date, there has been no record of successful delivery of the larger range of NAs, such as pDNA, via light-triggered endosomal escape. In this paper, we report on a first trial to do so, by making use of nanocomplexes composed of the cationic polymer JetPEI, pDNA and anionic 10 nm AuNP. In HeLa cells, we systematically evaluated the effect of heating vs. VNB formation on endosomal escape efficiency, pDNA integrity and final transfection efficiency. We demonstrate that laser-induced VNB are clearly more efficient in disrupting the endosomal membrane than mere heating. However, in the current JetPEI/pDNA/AuNPNP nanocomplex, the pDNA does not survive the highly intense physical forces when VNB are formed. On the other hand, while the generation of heat is less damaging to pDNA, endosomal escape is much less efficient. We conclude that a different design of AuNP containing nanocomplexes is needed that is still capable of forming VNB but that poses less stress on the pDNA.

## 2. Results

### 2.1. Synthesis and Characterization of JetPEI/pDNA/AuNP Complexes

JetPEI/pDNA/AuNP complexes were prepared as a vehicle to combine both pDNA and AuNP into a single complex to induce photothermally triggered endosomal escape of pDNA. JetPEI/pDNA complexes with a positive charge and an N/P ratio of 4 are mixed with negatively charged hyaluronic acid (HA) coated AuNPs. Both AuNPs as well as JetPEI/pDNA/AuNP complexes were characterized to ensure the formation of stable, reproducible complexes.

#### 2.1.1. Characterization of 10 nm AuNPs with HA (Hyaluronic Acid) Coating

**Figure 3:**
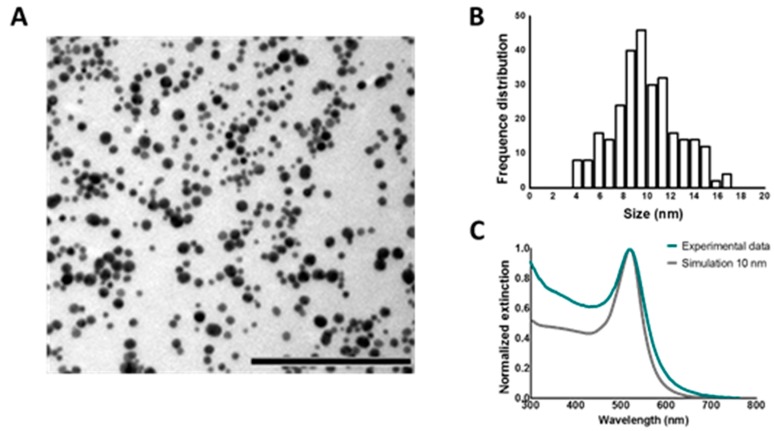
Characterization 10 nm AuNPs. (**A**) TEM (transmission electron microscopy) image of unfunctionalized AuNPs. Scalebar represents 200 nm; (**B**) size distribution of AuNP derived from TEM images; (**C**) normalized extinction spectrum of pristine AuNPs. The blue line represents the experimental data, as measured by UV/VIS spectrophotometry. The grey line represents the simulation of 10 nm AuNP according to the Mie theory.

First of all, 10 nm AuNPs were synthetized using ascorbate as a reducing agent. In order to verify that the AuNPs have a core size of 10 nm, characterization of these particles was performed. TEM images were recorded and are shown in Figure 3A. The core size of the NPs was determined via image processing and the result is displayed in Figure 3B. Next, the UV/VIS spectrum of the synthetized AuNPs was measured and compared to simulated data of the extinction of a 10 nm AuNP according to the Mie theory (see Figure 3C). Based on the obtained TEM size distribution and the excellent agreement between the experimental extinction spectrum to the simulated extinction cross-section spectrum, we conclude that the synthetized AuNPs have a core size of around 10 nm on average.

Functionalization of 10 nm AuNPs with HA was performed to allow complexation of the AuNPs to the positively charged JetPEI/pDNA complexes. Functionalization with HA was confirmed as the zeta potential of the AuNPs shifted from −14.7 ± 0.5 to −29.4 ± 3.21 mV (mean ± SEM) after addition of HA and subsequent centrifugation to remove unbound HA.

#### 2.1.2. Characterization of JetPEI/pDNA/AuNP Complexes

**Figure 4:**
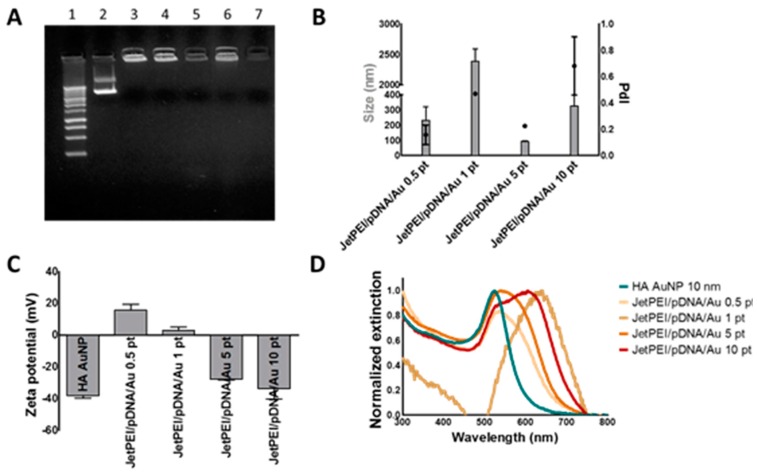
Characterization of JetPEI/pDNA/AuNP complexes. (**A**) gel electrophoresis shows successful pDNA complexation for all JetPEI/pDNA/AuNP complexes. Lane 1 shows a 1 kb ladder control. Lane 2 shows free pDNA. Lane 3 shows JetPEI/pDNA complexes prepared at an N/P charge ratio of 4. Lane 4, 5, 6 and 7 show JetPEI/pDNA/AuNP prepared with 0.5, 1, 5 and 10 pellets of AuNP, respectively. Further characterization of the complexes by dynamic light scattering reveals (**B**) the size (grey bars), PdI (black dots) and (**C**) Z potential. Values are displayed as mean ± stdev; *n* = 2; (**D**) normalized UV/VIS spectra of HA coated AuNPs (blue line) and JetPEI/pDNA/AuNP complexes (orange–red lines).

JetPEI/pDNA/AuNP complexes were prepared by mixing JetPEI/pDNA polyplexes with an N/P charge ratio of 4 with 10 nm HA coated AuNPs. AuNPs were first centrifuged to a pellet and added to the JetPEI/pDNA polyplexes in a range of concentrations: 0.5 pellets (0.5 pt; 7.2 × 10^10^ AuNPs), 1 pellet (1 pt; 1.44 × 10^11^ AuNPs), 5 pellets (5 pt; 7.2 × 10^11^ AuNPs) and 10 pellets (10 pt; 1.44 × 10^12^ AuNPs). These concentrations of AuNPs were calculated via the Lambert–Beer formula from UV/VIS measurements making use of the extinction coefficient provided by Mie theory calculations of 10 nm Au spheres (σ_520nm_ = 5.2 × 10^−13^ cm^2^/NP). Next, gel electrophoresis was performed to evaluate if pDNA was still retained inside the complexes. As can be seen from Figure 4A, the addition of HA coated AuNPs did not interfere with the complexation of pDNA to JetPEI. Next, hydrodynamic diameter, polydispersity index (PdI) and Z potential were determined using dynamic light scattering (DLS; Figure 4B,C). Finally, UV/VIS spectra of these complexes were measured, as can be seen in Figure 4D. JetPEI/pDNA/AuNP complexes prepared with 5 pt were selected for further experiments since they were able to complex pDNA and had the smallest size (92.5 ± 5.1 nm *n* = 3) with a negative zeta potential (−27.7 ± 0.5 mV *n* = 3). UV/VIS spectra showed that the plasmon peak of this complex is situated around 530–570 nm, which is well suited to absorb the 561 nm laser light of the pulsed laser that will be used for inducing photothermal endosomal escape. Finally, using Nanoparticle Tracking Analysis, the concentration of the JetPEI/pDNA/AuNP 5 pt complexes was measured to be 2.75 ± 0.35 × 10^11^ particles per mL.

### 2.2. Threshold Determination for Heating and VNB Formation

**Figure 5:**
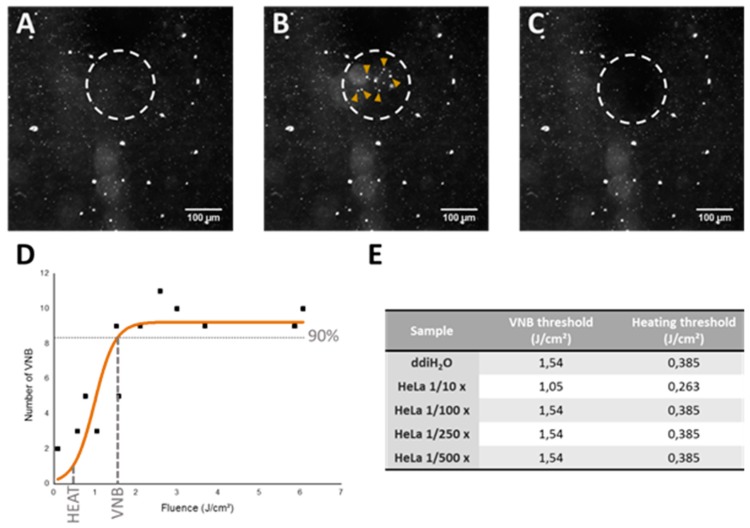
Determination of heating and VNB threshold via dark-field microscopy. (**A**) dark-field microscopy image of JetPEI/pDNA/AuNP 5 pt complexes in ddiH_2_O; (**B**) dark-field microscopy image upon VNB formation (VNBs indicated by yellow arrows); (**C**) dark-field microscopy image after VNB formation. Scalebar on the images represents 100 μm; (**D**) graph shows the relation between the number of VNBs and laser fluence. VNB threshold is calculated as the laser fluence needed to reach 90% of the maximum number of VNBs. The fluence for heating is selected at one-fourth of the VNB threshold; (**E**) the table shows the threshold values for VNB formation and heating (in J/cm^2^) used for JetPEI/pDNA/AuNP 5 pt complexes in further experiments.

In order to determine the laser fluence needed for heating or VNB formation, dark-field microscopy was performed since this method enables us to set up a graph to evaluate the effect of laser fluence on the formation of VNBs. As can be seen in Figure 5A–C, dark-field microscopy allows visualization of the AuNPs before, during and after VNB formation. In this way, the number of VNBs generated after a single pulse was counted and plotted against the used laser fluence. Afterwards, the VNB threshold is determined as the laser fluence at which VNBs are formed with 90% certainty and the fluence for heating is selected at one-fourth of the VNB threshold, in accordance with published literature [20]. An example of the graph used to determine heating and VNB thresholds in buffer can be seen in Figure 5D. As our purpose is to induce endosomal escape after endocytosis of the JetPEI/pDNA/AuNP complexes, similar experiments were performed after incubating HeLa cells with JetPEI/pDNA/AuNP 5 pt complexes for 1 h. Cells were incubated with different dilutions of the complexes in the range of 1/10 to 1/500. Apart from the highest concentration, the thresholds were nearly identical to those in water. Based on these results, the thresholds as displayed in Figure 5E were chosen for further experiments.

### 2.3. Evaluation of Uptake Efficiency

**Figure 6:**
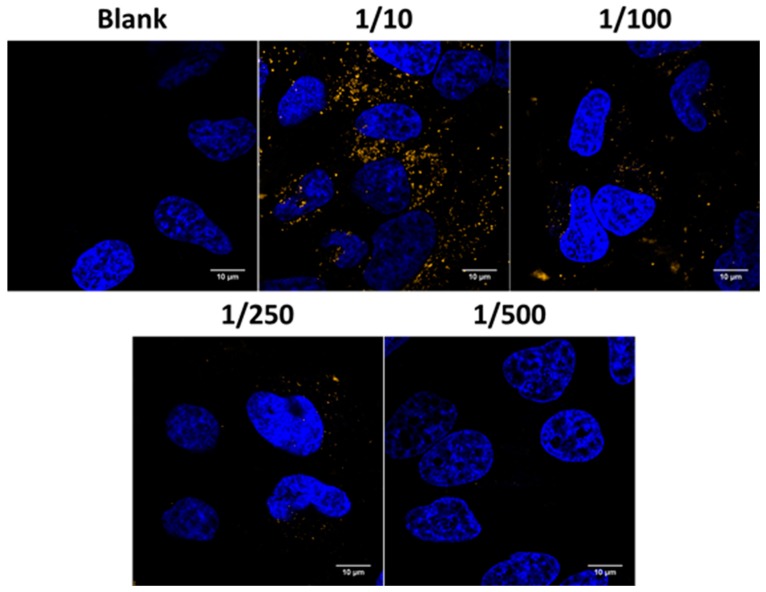
Evaluation of the uptake of JetPEI/pDNA/AuNP complexes in Hela cells. Confocal microscopy images show nuclei stained with Hoechst in the blue channel and AuNP core in the orange channel. The scale bar represents 10 μm.

Uptake experiments were carried out using confocal microscopy to visualize the uptake of JetPEI/pDNA/AuNP 5 pt complexes in HeLa cells. A representative image of each dilution is presented in Figure 6. The blue channel shows nuclei that were stained with Hoechst and the orange channel shows the complexes, which could be visualized in confocal reflection mode using the 561 nm laser because of its high scattering properties (due to the presence of AuNPs). Confocal images show that HeLa cells are capable of incorporating JetPEI/pDNA/AuNP 5 pt complexes. It must be noted, however, that the number of particles taken up after administration of a 1/500 dilution is very low, hinting that a further decrease in concentration likely would not be relevant.

### 2.4. Evaluation of Transfection Efficiency and Cell Viability

**Figure 7:**
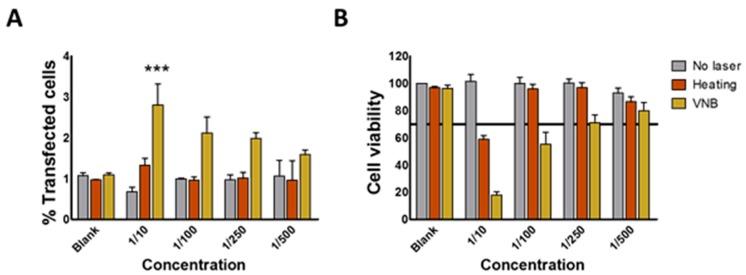
Evaluating transfection efficiency and cell viability in HeLa cells after laser irradiation in the heating or VNB regime. (**A**) the graph shows the percentage of cells that are positive for GFP transfection for different dilutions of JetPEI/pDNA/AuNP 5 pt complexes 24 h after laser irradiation; (**B**) the graph shows the corresponding percentage of viable cells as measured by DAPI staining. All graphs show mean ± SEM; *n* = 3. Significance was calculated using two-way ANOVA with a Bonferroni post-test (compare means to blank) (*** *p* < 0.001).

Next transfection efficiencies of the JetPEI/pDNA/AuNP 5 pt complexes was evaluated upon laser irradiation, together with cellular toxicity. GFP (green fluorescence protein) expression was evaluated after 24 h via flow cytometry. As shown in Figure 7A, the complexes by themselves (i.e., without laser irradiation) showed no transfection at any of the concentrations tested, as expected for the selected N/P charge ratio of 4. Cells irradiated with a low intensity laser pulse (heating regime) were not transfected either. At high laser pulse energy (VNB regime), a small fraction of the cells (3%) became transfected in case of the highest concentration of complexes. Cell viability was measured in parallel by DAPI (2-(4-Amidinophenyl)-6-indolecarbamidine dihydrochloride) staining. DAPI is excluded from viable cells, while it is able to enter into non-viable cells whose cell membrane integrity is compromised. Non-viable cells become positive for DAPI fluorescence, which increases dramatically upon DNA intercalation. The DAPI signal is quantified by flow cytometry alongside the GFP signal. Experiments showed acceptable cell viability (>70%) in the heating regime starting from a 1/100 dilution and in the VNB regime starting from a 1/250 dilution (see Figure 7B). Unfortunately, no significant transfection was observed for those conditions. In spite of non-successful transfections, it is of fundamental interest to try to find out why they were unsuccessful as these insights might teach us how to improve future systems for light-triggered pDNA transfections.

### 2.5. Evaluation of Endosomal Escape and pDNA Breakdown

**Figure 8:**
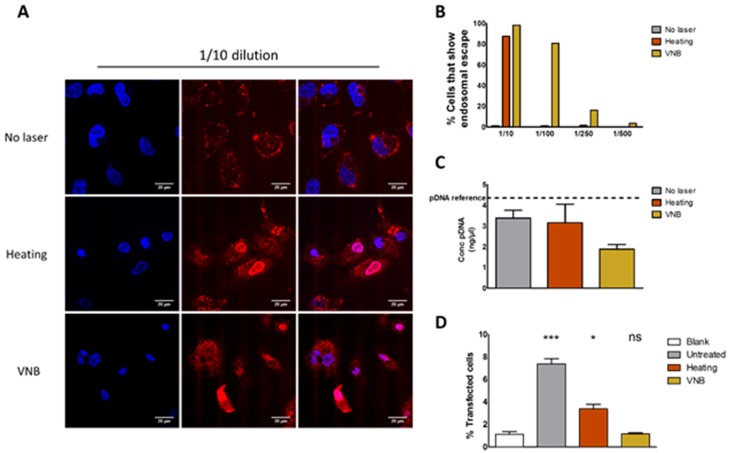
Evaluation of endosomal escape and pDNA integrity. (**A**) confocal images show the result of the endosomal escape assay without laser treatment, after heating and after VNB formation of JetPEI/pDNA/AuNP 5 pt complexes into which fluorescently labeled oligonucleotides (ONs) were co-complexed. Upon successful endosomal escape, the fluorescent ONs accumulate into the nucleus. The scalebar represents 20 μm. The left column shows nuclei after Hoechst staining; the middle column shows AF647 ONs; and the right column shows the merge. (**B**) from confocal images, the percentage of cells is calculated that show endosomal escape (red nucleus) for the different dilutions of JetPEI/pDNA/AuNP 5 pt complexes. The data is obtained from the analysis of 60–100 cells per condition; (**C**) the pDNA concentration was measured via PicoGreen assay after the addition of dextran sulphate. The dotted line represents the amount of pDNA originally added to the complexes (pDNA reference). Graph shows mean ± SEM; *n* = 2; (**D**) graph shows the percentage of transfection efficiency after electroporation with isolated pDNA from JetPEI/pDNA/AuNP complexes. (*** *p* < 0.0001; * *p* < 0.05; ns = not significant).

A first possible reason why transfections were unsuccessful is a lack of endosomal escape upon laser irradiation. Therefore, endosomal escape efficiency was investigated specifically by time-lapse confocal microscopy. This was achieved by incorporating fluorescently labeled oligonucleotides (AF647 ONs) into the complexes that accumulate into the nucleus upon endosomal release [30,31]. Thus, cells in which endosomal escape happened can be quantified by counting red nuclei in confocal microscopy images. After an incubation period of 1 h, the cells underwent laser treatment and were allowed to stabilize for 3 h. Next, cells were visualized by confocal microscopy and the number of red nuclei (indicative for endosomal escape) was counted. Figure 8A shows confocal images of the 1/10 concentration and illustrate that, for this concentration, endosomal escape occurred in nearly every cell. The percentage of cells that showed endosomal escape is displayed in Figure 8B as a function of complex concentration and indicates that the VNB regime is more efficient in inducing endosomal escape than the heating regime. We conclude that photothermally induced rupture of endosomes was successful, as evidenced by the release of fluorescent oligonucleotides, and that VNB induced endosomal escape is by far the most efficient.

Another reason why transfections were unsuccessful could be because the pDNA is damaged by the laser-induced photothermal effects. To investigate the effect of heating and VNB formation on the structure of pDNA, a PicoGreen assay was performed. To this end, JetPEI/pDNA/AuNP 5 pt complexes were irradiated with low (heat) or high (VNB) intensity laser pulses, and the amount of pDNA remaining after laser irradiation was measured by the use of a PicoGreen assay. PicoGreen becomes fluorescent upon DNA intercalation, which can only happen after it has been released from the complexes. Therefore, we added dextran sulphate to the complexes after laser irradiation to release the remaining pDNA and check if it is still intact. The concentration of pDNA released from the complexes and measured by PicoGreen assay can be seen in Figure 8C. In case of VNB treatment, it was noted that the remaining pDNA was only half the amount that was originally incorporated into the complexes. This drop indicates the degradation of a substantial amount of pDNA in such a way that PicoGreen is no longer able to intercalate with the remains.

The fact that (some) pDNA structure is still detectable via PicoGreen assay does not necessarily mean that the remaining pDNA is intact and functional. Indeed, it could be possible that the remaining pDNA is fragmented after laser treatment, still allowing PicoGreen intercalation but unable to produce transfection. To determine the functionality of the remaining pDNA after laser treatment, we evaluated the transfection of HeLa cells with this remaining pDNA through electroporation (Figure 8D). As above, complexes were irradiated with laser pulses and treated with dextran-sulfate to release all pDNA. This solution was then added to the cells, which subsequently underwent electroporation. Results showed that electroporation of HeLa cells with pDNA, isolated from complexes that underwent heating (i.e., irradiation with low laser intensity), resulted in a transfection efficiency that was significantly higher than the blank, but lower than the pDNA from non-irradiated complexes. This indicates that, even though a substantial fraction of pDNA became damaged, there is at least some functional pDNA left. VNB treatment of the complexes (i.e., irradiation at high laser intensity), on the other hand, could not produce any transfection after electroporation, showing that VNB treatment completely disturbs the functionality of the pDNA in the JetPEI/pDNA/AuNP complexes.

## 3. Discussion

In recent years, the interest in AuNPs for drug delivery purposes has increased tremendously, not in the least because their optical properties could be used in order to obtain spatio-temporally controlled delivery of cargo. In this article, we aimed to use the plasmonic properties of AuNPs to induce light triggered rupture of the endosomal membrane after incubation of HeLa cells with JetPEI/pDNA/AuNP complexes. We examined if photothermal effects such as heating and VNB formation were able to induce endosomal release and transfect HeLa cells. After evaluation of JetPEI/pDNA/AuNP complexes prepared with different amounts of AuNPs, JetPEI/pDNA/AuNP 5 pt complexes were selected for further experiments since they showed a good size, zeta potential and UV/VIS extinction spectrum. Even though the complexes were taken up by cells, unfortunately, we observed that neither heat transfer nor VNB formation were able to induce any meaningful transfection.

In order to find out what the cause of this inefficient transfection is, we first studied endosomal escape through the use of an ON dequenching assay. Results showed that both heat transfer and VNB formation were able to induce endosomal escape, with VNB being by far the most efficient of the two. We hypothesize that, in order to induce endosomal escape via heating, a relatively high concentration of AuNPs is needed per endosome in order to heat up the endosomal lumen to a temperature that is high enough to induce thermal membrane destabilization. On the other hand, the mechanical force arising from the formation of already one VNB is likely sufficient to induce mechanical disruption of the endosomal membrane and thus release endosomal content. Two side notes must be made on the use of the ON dequenching assay to evaluate endosomal escape. First of all, it should be kept in mind that the endosomal release of the small ONs might not be fully representative to evaluate endosomal escape of a much larger construct such as pDNA. While it shows that the endosomal membrane was permeabilized, it remains uncertain if larger pDNA molecules can escape from the endosomes—especially in the case of heat-induced permeabilization pores possibly being on the small side, as already known from plasma membrane photoporation [20]. Secondly, low concentrations of complexes had to be used to keep toxicity at an acceptable level. When using these low concentrations, visual confirmation of endosomal escape based on the ON dequenching assay after VNB formation became difficult. If the amount of AF647 ONs per endosome is not high enough to be detected, endosomal escape could remain invisible using this method, despite the fact that it may actually have happened. In order to confirm our hypothesis that one VNB is capable of disrupting the endosomal membrane, it would be useful to use a more sensitive assay to detect endosomal escape. Such an assay was proposed by Wittrup et al. where they used two different exposure settings in order to extend the dynamic range. Using long exposure times, weakly fluorescent signals that may remain unnoticed in the dequenching assay should become detectable [32]. Nevertheless, since, in the 1/10 dilution, the majority of cells show endosomal escape after VNB formation and after heat transfer, we conclude that the endosomal rupture is not the limiting factor for photothermally triggered transfection.

We next went on to evaluate the integrity of pDNA after inducing the photothermal effects. Mechanical or thermal stimuli may damage nucleic acids, rendering the pDNA ineffective even when it is able to reach the cytosol. Although photothermally triggered endosomal escape of siRNA has been performed successfully [15,28,29], pDNA offers an extra challenge because of its much longer sequence. Therefore, we examined the pDNA content after VNB formation and heating regime via PicoGreen assay. We found that a large part of the cargo was degraded after VNB formation as PicoGreen was no longer able to intercalate. However, there was still a considerable amount of structural pDNA detected after heating and VNB formation. Since a PicoGreen assay only indicates the presence of pDNA structure, it does not provide any information regarding the integrity (and thus transfection potential) of the remaining pDNA. Therefore, we evaluated the functionality of pDNA after release from the (laser treated) complexes by dextran sulphate. HeLa cells were transfected with this released pDNA by electroporation. We found that the formation of VNBs damages the pDNA completely in JetPEI/pDNA/AuNP complexes, thereby abolishing the potential for transfection. In the case of heating, part of the pDNA survived. In spite of that, no transfection was observed, which points to the fact that heat-induced pores in the endosomal membrane are likely not large enough for pDNA to pass through, as already mentioned above.

**Figure 9:**
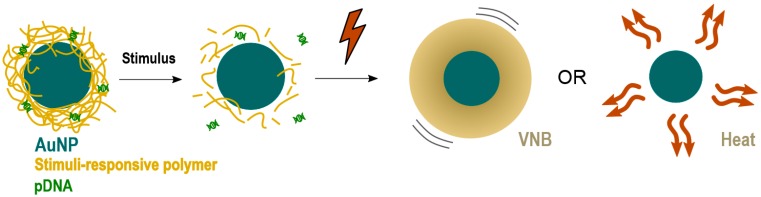
Alternative NP design for cytosolic delivery of intact pDNA after endocytosis.

The results presented here demonstrate that VNBs can rupture endosomes. Nevertheless, the current design must be improved in order to be able to generate transfections’ efficiencies comparable to benchmarks techniques like lipofectamine, which show a transfection efficiency of 15% for an equal amount of pDNA as used for JetPEI/pDNA/AuNP complexes in the 1/10 condition (Figure S1). To reach this goal, an NP has to be designed that better protects the pDNA against these photothermal effects. One option would be to design an NP that has a gold core, surrounded by a stimuli-responsive polymer that allows the release of pDNA prior to photothermal laser treatment, as shown in Figure 9. Such stimuli-responsive polymers could be pH-responsive polymers that degrade upon endosomal acidification or thermo-sensitive polymers that degrade after an initial soft heating step. By making sure that the pDNA is dissociated from the carrier before laser treatment, pDNA is expected to better survive the subsequent irradiation step to induce endosomal escape.

## 4. Materials and Methods

### 4.1. Materials

DMEM (Dulbecco’s Modified Eagle Medium)/F-12, l-Glutamine, Penicillin-Streptomycin solution (5000 IU/mL penicillin and 5000 μg/mL streptomycin) (P/S), Fetal Bovine Serum (FBS), Opti-MEM, Trypan Blue, 0.25% Trypsin-EDTA (ethylenediaminetetraacetic acid) and Dulbecco’s phosphate-buffered saline 1× without Ca^2+^/Mg^2+^ (DPBS-) were provided by GibcoBRL (Merelbeke, Belgium). YOYO-1 iodide, Hoechst 33342 and Quant-IT PicoGreen dsDNA Assay Kit were supplied by Molecular Probes (Erembodegem, Belgium). Other reagents were purchased from Sigma-Aldrich (Bornem, Belgium) unless otherwise specified.

### 4.2. Synthesis and Characterization of Hyaluronic Acid Coated 10 nm Gold Nanoparticles

#### 4.2.1. Synthesis of 10 nm Gold Nanoparticles

The synthesis of 10 nm gold nanoparticles (AuNPs) was performed using ascorbate as reducing agent. A typical synthesis consists of adding Au to give a final concentration of 0.2 mM HAuCl_4_ with the addition of equimolar quantities of sodium ascorbate (final volume = 100 mL) under rapid stirring and let react for 30 min.

The characterization of AuNPs was performed combining UV/VIS spectroscopy, dynamic light scattering (DLS), transmission electron microscopy (TEM), and electrodynamic modeling using Mie theory. UV/VIS spectroscopy was performed on a NanoDrop 2000c spectrophotometer (Thermo Scientific, Rockford, IL, USA). DLS measurements were carried out using a Zetasizer Nano (Malvern, Worcestershire, UK) and disposable folded capillary cells (Malvern, Worcestershire, UK) to determine hydrodynamic diameter, polydispersity index and zeta potential. TEM images were obtained at the VIB-UGent Transmission Electron Microscopy-Core facility using a JEM 1400 plus transmission electron microscope (JEOL, Tokyo, Japan) operating at 60 kV. Samples were prepared by adding one drop (~50 μL) of the samples colloidal solution onto formvar/C-coated hexagonal copper grids (EMS G200H-Cu) for 20 min and washed 5 times in double distilled water (ddiH_2_O). Finally, the size and concentration of AuNPs was estimated using the experimental extinction intensities at the maximum wavelength (λ_max_ = 520 nm), and Mie theory calculations [33,34,35] of the extinction cross section for spherical particles (σ_ext_(520 nm) = 5.2 × 10^−13^ cm^2^/NP).

#### 4.2.2. Functionalization with HA to Form HA AuNP 10 nm

The synthetized NPs were immediately functionalized with hyaluronic acid (HA) to install a negative Z potential, required for complexation with positively charged JetPEI/pDNA complexes. Typically, functionalization with HA was performed by adding 6–10 mg of the polymer (all stock solutions of the synthetized AuNPs in pM concentration). Successful functionalization was confirmed by DLS Z potential measurements, performed after centrifugation of the AuNPs to remove unbound HA.

### 4.3. Preparation of Plasmids

gWIZ GFP (Promega, Leiden, The Netherlands) was amplified in transformed *E. coli* bacteria and isolated from this bacteria suspension using a Qiafilter Plasmid Giga Kit (Qiagen, Venlo, The Netherlands). Concentration was determined on a NanoDrop 2000c (Thermo Fisher Scientific, Rockford, IL, USA) by UV absorption at 260 and 280 nm and adjusted to a final concentration of 1 μg/μL with HEPES buffer (20 mM, pH 7.2).

### 4.4. Preparation of Au Functionalized JetPEI/pDNA Nanoparticles

JetPEI/pDNA polyplexes were prepared using commercially available JetPEI (Polyplus transfection, Leusden, The Netherlands). JetPEI/pDNA complexes were obtained by mixing the polymer solution with an equal volume of pDNA solution. N/P ratio of the polyplexes was calculated using the formula provided by the manufacturer (Equation (1)). Next, the mixture was vortexed for 10 s at 2200 rpm and polyplexes were allowed to stabilize for 15 min:

(1) $$N/P\;\mathrm{ratio}=\frac{7.5\;x\;\mu L\;\mathrm{of}\;\mathrm{JetPEI}}{3\;x\;\mu g\;\mathrm{of}\;\mathrm{DNA}}$$

Next, the required amount of HA coated 10 nm AuNPs was centrifuged at 12,000× *g* for 10 min. The supernatant was removed and the pellet was resuspended in ddiH_2_O before mixing with JetPEI/pDNA complexes in equal volumes. The resultant mixture was allowed to stabilize for 30 min followed by final dilution with ddiH_2_O.

### 4.5. Physicochemical Characterization of JetPEI/pDNA/AuNP Complexes

To evaluate complexation of pDNA to the JetPEI/pDNA/AuNP complexes, gel electrophoresis was performed. The complexes were prepared as described above. A 1% agarose gel was prepared by dissolving 1 g of agarose (UltraPure Agarose, Invitrogen, Erembodegem, Belgium) in 100 mL of 1× Tris/Borate/EDTA (TBE) buffer after which GelRed (Biotium, Hayward, CA, USA) was added in order to detect pDNA. In addition, 5 μL of Gel Loading Buffer (Ambion, Merelbeke, Belgium) was added to 20 μL of complexes and a total volume of 25 μL was pipetted in every lane. A 1 kb ladder (Bioron GmbH, Ludwigshafen, Germany) and uncomplexed pDNA were taken along as controls. Gel electrophoresis was performed at 100 V for 30 min and a PhotoDoc-It Imaging system (Upland, CA, USA) was used to acquire an image of the gel under UV light (Bio-Rad UV transilluminator 2000, Hercules, CA, USA).

Next, Dynamic Light Scattering measurements were performed on the NanoZS Zetasizer. The complexes were prepared as described above and were transferred to disposable folded capillary cells to determine hydrodynamic diameter, polydispersity index and zeta potential. The same complexes were used to measure the UV/VIS spectrum on a NanoDrop 2000c. Finally, the concentration of JetPEI/pDNA/AuNP complexes was measured via Nanoparticle Tracking Analysis using the NanoSight LM10 (Malvern, Worcestershire, UK). The measurements were performed in quintuplet.

### 4.6. Cell Culture

HeLa cells (cervical adenocarcinoma cells, ATCC CCL-2) were cultured in Dulbecco’s modified Eagle’s medium supplemented with growth factor F12 (DMEM/F-12) and enriched with 10% FBS, 2 mM l-Glutamine and 100 μg/mL P/S. Cells were cultured in a humidified atmosphere at 37 °C and 5% CO_2_. Experiments were performed on cells with a passage number below 25.

### 4.7. Generation and Detection of JetPEI/pDNA/AuNP Complex Heating and Vapour Nanobubble (VNB) Formation

**Figure 10:**
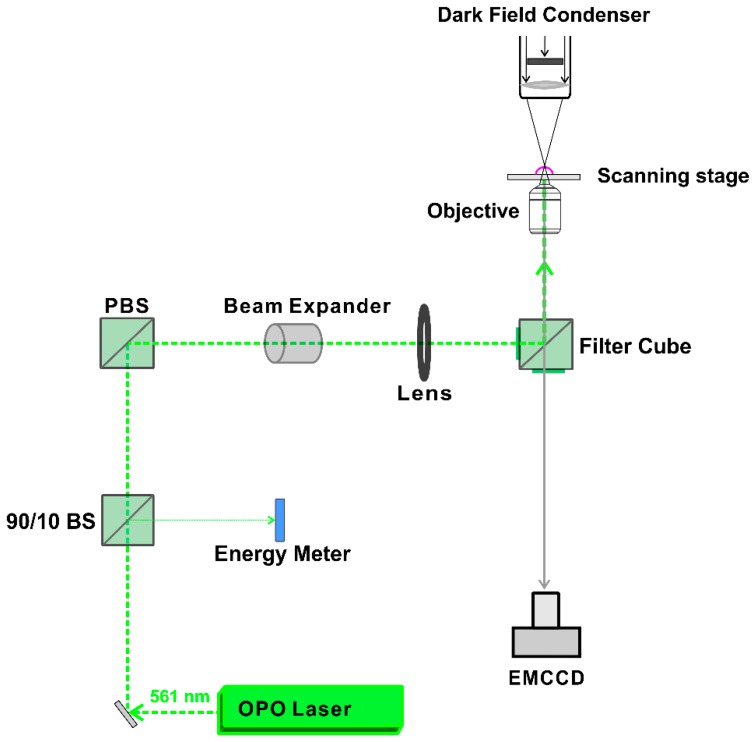
Optical design for generation and detection of heating and VNB formation. AOTF: acousto-optic modulator to control the power of the continuous wave laser. OPO laser: pulsed laser with ~7 ns pulses equipped with an Optic Parametric Oscillator that allows for tuning the wavelength from 410 to 2200 nm. 90/10 BS: laser beam splitter that reflects 10% and transmits 90% of the laser light. PBS: polarization beam splitter. Image adjusted with permission from [20] © American Chemical Society.

A homemade setup, according to the optical design in Figure 10, was used to generate and detect heating or VNB formation. The setup is built around an inverted TE2000 epi-fluorescence microscope (Nikon, Nikon BeLux, Brussels, Belgium) equipped with a Plan Fluor 10 × 0.3 NA lens (Nikon, Tokyo, Japan). A pulsed laser with a pulse duration of ~7 ns was tuned at a wavelength of 561 nm with an Optical Parametric Oscillator (OPO) laser (Opolette™ HE 355 LD, OPOTEK Inc., Carlsbad, CA, USA) and used to excite the localized surface plasmon resonance of the JetPEI/pDNA/AuNP complexes. The energy of the laser pulses was measured with an energy meter (J-25MB-HE&LE, Energy Max-USB/RS sensors, Coherent, Santa Clara, CA, USA).

Detection of VNB formation was performed using dark-field microscopy as VNBs efficiently scatter light. Since VNBs typically have a very short lifetime (<1 μs), the camera (EMCCD camera, Cascade II: 512, Photometrics, Tucson, AZ, USA) was synchronized with the pulsed laser using an electronic pulse generator (BNC575, Berkely Nucleonics Corporation, San Rafael, CA, USA). The pulse laser sends a Q-switch signal to trigger the pulse generator and the camera at a certain delay. In this way, dark-field images were taken before, during and after illumination. Dark-field microscopy was used to determine the fluence threshold for VNB formation and heating of JetPEI/pDNA/AuNP complexes. To determine the thresholds in HeLa cells, cells were first seeded in 50 mm γ-irradiated glass bottom dishes (MatTek Corporation, Ashland, MA, USA) at a density of 600,000 cells. Cells were allowed to attach overnight and the next day, complexes were added to the cells in Opti-MEM. After incubation at 37 °C for 1 h, the cells were washed, full culture medium was added and dark-field microscopy was performed. In order to calculate the threshold for heating and VNB formation, dark-field images were analyzed using ImageJ (https://fiji.sc/) and the number of VNBs was plotted in function of the laser fluence that was used.

For scanning larger areas such as wells of a 96-well plate, we used an automatic Prior Proscan III stage (Prior Scientific Ltd., Cambridge, UK) to scan the sample line by line with a scanning speed of 2.2 mm/s and a 150 μm diameter laser beam with 20 Hz pulse frequency. The distance between subsequent lines was set to 110 μm to ensure the illumination of all complexes present in the sample.

### 4.8. Evaluation of Transfection Efficiency and Cytotoxicity

HeLa cells were seeded in 96-well plates at 10,000 cells per well and were allowed to attach overnight. The next day, JetPEI/pDNA/AuNP complexes containing gWIZ GFP were prepared as described above. Cells were incubated with JetPEI/pDNA/AuNP complexes in Opti-MEM for 1 h at 37 °C. Afterwards, they were washed with Opti-MEM and full cell culture medium was added before laser treatment, as described above. After laser treatment, the cells were cultured for another 24 h before they were prepared for flow cytometry analysis. To examine transfection efficiency, expression of gWIZ GFP was measured in the green channel. For cytotoxicity evaluation, DAPI (Thermofischer, Waltham, MA, USA) was added to the flow buffer according to the manufacturer’s instructions.

Additionally, the results obtained with JetPEI/pDNA/AuNP complexes were compared with a positive control using Lipofectamine 2000 (Invitrogen, Carlsbad, CA, USA) following the manufacturer’s instructions and using the same final concentrations as for the JetPEI/DNA complexes. 

### 4.9. Flow Cytometry

To perform analysis by flow cytometry, cells were detached using trypsin and transferred to flow cytometry tubes (BD Falcon, Radnor, PA, USA). Next, the cell suspensions were centrifuged at 300× *g* for 5 min (Bio-Rad DiaCent-12, DieMed GmbH, Cressier, Switzerland) and resuspended in flow buffer (DPBS-, 0.1% Sodium Azide, 1% Bovine Serum Albumine). Finally, samples were vortexed at 2200 rpm (YellowLine TTS2, IKA works, Wilmington, DC, USA). Flow cytometry was performed on 10,000 events per sample (CytoFLEX^TM^ Flow Cytometer, Beckman Coulter, Krefeld, Germany) or for a total duration of 120 s. gWIZ GFP fluorescence was detected with 525/40 nm bandpass filter after 488 nm excitation. DAPI fluorescence was detected with a 450/45 nm bandpass filter after 405 nm excitation. FlowJo software (version, Treestar Inc., Ashland, OR, USA) was used to perform the analysis.

### 4.10. Evaluation of Uptake Efficiency

Cells were seeded in 96-well plates with glass bottom (Greiner Bio-One, Frickenhausen, Germany) at a density of 10,000 cells per well and were allowed to attach overnight. The next day, cell nuclei were stained with Hoechst 33342 staining (1 mg/mL in H_2_O; 1000× diluted). JetPEI/pDNA/AuNP 5 pt complexes were prepared as described above. Cells were incubated with JetPEI/pDNA/AuNP 5 pt complexes in Opti-MEM for 1 h at 37 °C. After washing the particles off, the cells were provided with full cell culture medium and live-cell imaging was performed using a confocal laser scanning microscope (C1si, Nikon, Tokyo, Japan). A Plan Apo VS 60× 1.4 NA oil immersion objective lens (Nikon, Tokyo, Japan) was used to obtain a pixel size of 70 nm and AuNP were detected by the reflected laser light of the 561 nm laser. Image processing was performed using ImageJ (FIJI) software.

### 4.11. Visualization and Quantification of Endosomal Escape

Visualization and quantification of endosomal escape was performed based on the dequenching assay that was first published by Rehman et al. [30] Therefore, red-fluorescent oligonucleotides (AF647 ONs) were co-incorporated into the complexes. Cells were seeded in 96-well plates with glass bottom at a density of 10,000 cells per well and were allowed to attach overnight. Cell nuclei were stained with Hoechst 33342 staining (1 mg/mL in H_2_O; 1000× diluted). Next, AF647 ON-containing complexes were added to the cells in Opti-MEM and incubated for 1 h at 37 °C. After washing off the complexes, the cells were provided with full cell culture medium and laser treatment was performed, as described above. After laser treatment, the cells were imaged using a spinning disk confocal (SDC) microscope (Nikon Eclipse Ti, Tokyo, Japan) equipped with an MLC 400 B laser box (Agilent Technologies, Santa Clara, CA, USA), a Yokogawa CSU-X confocal spinning disk device (Andor, Belfast, UK), an iXon ultra EMCCD camera (Andor Technology, Belfast, UK) and NIS Elements software (Nikon, Japan). A Plan Apo VC 60× 1.4 NA oil immersion objective lens (Nikon, City, Japan) was used to yield an image pixel size of 234 nm. Exposure time was set to 20 ms and the images were processed using ImageJ (FIJI).

### 4.12. Determination of pDNA Integrity

#### 4.12.1. PicoGreen Assay to Evaluate pDNA Structure

JetPEI/pDNA/AuNP 5 pt complexes were prepared and underwent laser treatment, as described above. To the resulting samples (untreated, heat and VNB treated), dextran sulphate (50 mg/mL in PBS-) was added in order to release the complexed pDNA. Finally, the amount of pDNA in the samples was quantified using Quant-IT PicoGreen dsDNA Assay Kit according to the manufacturer’s protocol. Fluorescent measurements of the assay were performed on a fluorescence microplate reader (Tecan, Mechelen, Belgium).

#### 4.12.2. Electroporation to Evaluate pDNA Functionality

JetPEI/pDNA/AuNP 5 pt complexes were prepared and underwent laser treatment, as described above. To the resulting samples (untreated, heat and VNB treated), dextran sulphate (50 mg/mL in PBS-) was added in order to release the complexed pDNA. In order to evaluate the functionality, HeLa cells were electroporated in the presence of this released pDNA. In addition, 500,000 HeLa cells were suspended in 90 μL SE cell line solution (Lonza Cologne GmbH, Cologne, Germany) and 10 μL of the corresponding samples (containing an originally complexed amount of 17 ng gWIZ GFP pDNA) was added to the cells. Electroporation was performed using an Amaxa 4D-Nucleofector in HeLa cells, according to the manufacturer’s protocol (Lonza Cologne GmbH, Cologne, Germany). Next, samples were diluted with cell culture medium and seeded in 96-well plates at a density of 15,000 cells. The cells were incubated for 24 h and were then prepared for analysis via flow cytometry to evaluate transfection efficiency.

## 5. Conclusions

Photothermally triggered endosomal escape of pDNA is a fascinating technique that could be used to increase endosomal escape capacity of pDNA complexes. The results obtained in this paper show that, although this technique has been used before to induce endosomal escape of siRNA, the delivery of much larger pDNA poses extra challenges. Unfortunately, we observed that neither the formation of VNBs nor the generation of heat were able to induce efficient transfection in HeLa cells. The main reason for this lack of transfection is that, after VNB formation, the pDNA becomes dysfunctional, presumably due to damage by the physical strain imposed by the violent formation and collapse of VNB. For heat generation, even though a fraction of the pDNA remains intact, it is hypothesized that the pores formed in the endosomal membrane are not big enough to allow endosomal escape of large macromolecules such as pDNA. In the future, improvements to the design of the complex are needed to ensure endosomal release of intact pDNA.

## Supplementary Materials

Supplementary materials can be found at http://www.mdpi.com/1422-0067/19/8/2400/s1.
