# Supplementary material for: Photothermally Triggered Endosomal Escape and Its Influence on Transfection Efficiency of Gold-Functionalized JetPEI/pDNA Nanoparticles
Source: Int J Mol Sci. 2018 Aug 14;19(8):2400. doi: 10.3390/ijms19082400 (PMC6121899; doi:10.3390/ijms19082400)
Supplement: Supplementary file 1 [file ijms-19-02400-s001.pdf]

Supplementary Materials

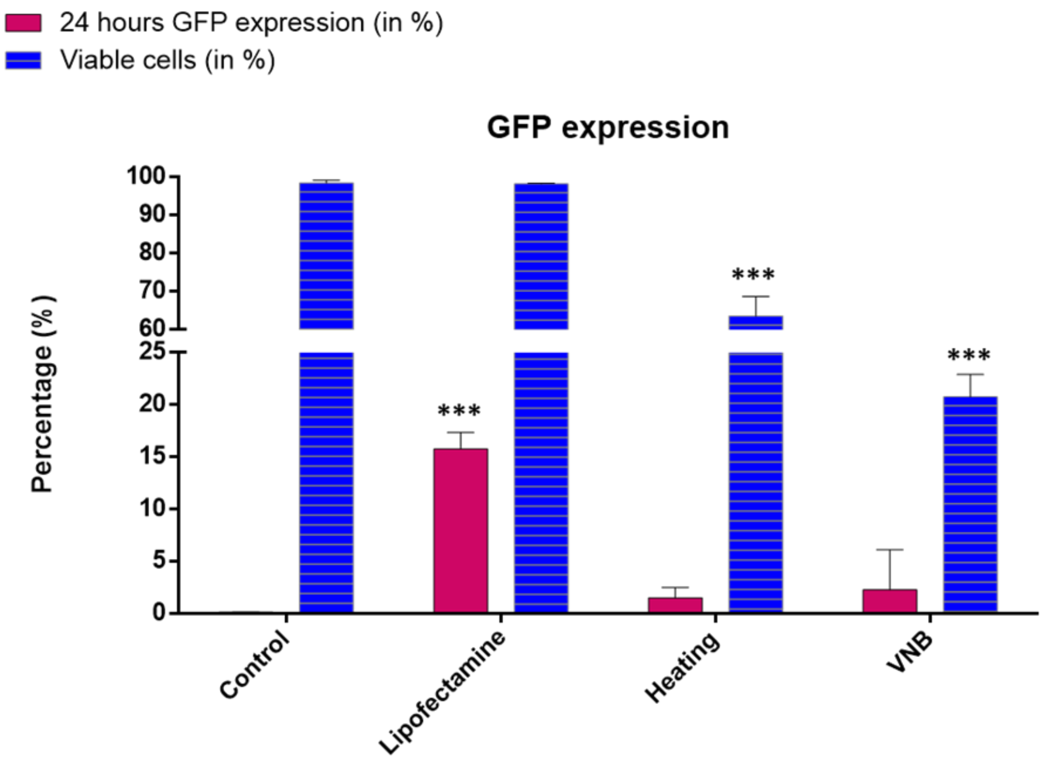

Figure S1. Evaluating transfection efficiency and cell viability in HeLa cells with Lipofectamine 2000. The graph shows the percentage of cells that are positive for GFP and the percentage of viable cells as measured by DAPI staining after 24h after. The concentration of pDNA used for lipofectamine experiments correspond to an equal amount as used for JetPEI/pDNA/AuNP complexes in the 1/10 condition. The data are shown as mean ± SEM; n=3. Significance was calculated using two-way ANOVA with Bonferroni post-test (*** $p < 0.001$).
